# Training and Experience of Peer Reviewers: Authors' Reply

**DOI:** 10.1371/journal.pmed.0040145

**Published:** 2007-03-27

**Authors:** Michael Callaham

Dr. Ignacio García-Doval [1] raises an interesting question—if a reviewer initially proves themselves of high quality at a particular journal, can we count on them to continue in this vein?

We have not examined this as thoroughly as the predictive factors, but we have found that good reviewers, on average, continue to produce good reviews for many years. However, their performance is not so consistent that one can completely cease monitoring them, because a modest proportion will deteriorate, presumably due to changes in their personal or professional lives. We have had reviewers who were reliably good for many years, but whose scores then steadily deteriorated until we were forced to retire them.

This reinforces our recommendation that all but the most resource-poor journals should routinely rate reviewers, something not hard to do in this era of ubiquitous computer databases. We recommend a look at those ratings about once a year, and feedback of some kind to those doing poorly. Another benefit is that this also identifies the high performers, who can then be rewarded in some way for their donated labor (a note of thanks, inexpensive objects with your journal logo, free continuing medical education for their review time, etc.). Consistent very high performers also serve as a good source for future editorial board appointments.
